# Tumor Biomarker Testing for Metastatic Colorectal Cancer: a Canadian Consensus Practice Guideline

**DOI:** 10.1177/17588359221111705

**Published:** 2022-07-20

**Authors:** Irene S. Yu, Francine Aubin, Rachel Goodwin, Jonathan M. Loree, Cheryl Mather, Brandon S. Sheffield, Stephanie Snow, Sharlene Gill

**Affiliations:** Department of Medical Oncology, BC Cancer Surrey, Surrey, BC, Canada; Division of Hematology and Oncology, Department of Medicine, Centre Hospitalier de l’Université de Montréal, Montreal, QC, Canada; Division of Medical Oncology, Department of Medicine, Ottawa Hospital Cancer Centre, Ottawa, ON, Canada; Department of Medical Oncology, BC Cancer Agency - Vancouver Centre, Vancouver, BC, Canada; Department of Laboratory Medicine and Pathology, University of Alberta, Edmonton, AB, Canada; Division of Advanced Diagnostics, William Osler Health System, Brampton, ON, Canada; Department of Medicine, Queen Elizabeth II Health Sciences Centre, Halifax, NS, Canada; Department of Medical Oncology, BC Cancer Agency – Vancouver Centre, 600 W 10th Ave, Vancouver, BC, V5Z 4E6, Canada

**Keywords:** colorectal cancer, metastasis, molecular testing, predictive biomarker, targeted therapy

## Abstract

The systemic therapy management of metastatic colorectal cancer (mCRC) has evolved from primarily cytotoxic chemotherapies to now include targeted agents given alone or in combination with chemotherapy, and immune checkpoint inhibitors. A better understanding of the pathogenesis and molecular drivers of colorectal cancer not only aided the development of novel targeted therapies but led to the discovery of tumor mutations which act as predictive biomarkers for therapeutic response. Mutational status of the *KRAS* gene became the first genomic biomarker to be established as part of standard of care molecular testing, where *KRAS* mutations within exons 2, 3, and 4 predict a lack of response to anti- epidermal growth factor receptor therapies. Since then, several other biomarkers have become relevant to inform mCRC treatment; however, there are no published Canadian guidelines which reflect the current standards for biomarker testing. This guideline was developed by a pan-Canadian advisory group to provide contemporary, evidence-based recommendations on the minimum acceptable standards for biomarker testing in mCRC, and to describe additional biomarkers for consideration.

## Introduction

Colorectal cancer (CRC) is the third most commonly diagnosed cancer in Canada and worldwide, accounting for approximately 10% of all cancer diagnoses.^1,2^ Mortality rates for CRC have continued to decline over the past 40 years, which has likely been driven by implementation of cancer screening programs and access to improved therapies. However, 5-year survival rates remain at 67%, with rates as low as 11% for those with stage IV disease at diagnosis.^3^ Approximately 20% of patients with newly diagnosed CRC present with metastases and an additional 50% of patients initially diagnosed with stage I–III disease will progress to metastatic disease, where surgical control is difficult.^4^

Chemotherapy remains the backbone for management of metastatic CRC (mCRC), consisting of a combination of fluoropyrimidine agents with either irinotecan [5-fluorouracil, leucovorin, irinotecan (FOLFIRI)] or oxaliplatin [5-fluorouracil, leucovorin, oxaliplatin (FOLFOX)]. Over the last 20 years, several therapies targeting pathways that contribute to mCRC pathogenesis entered the treatment paradigm, including monoclonal antibodies and tyrosine kinase inhibitors against the epidermal growth factor receptor (EGFR; cetuximab and panitumumab), vascular endothelial growth factor (VEGF; bevacizumab, regorafenib, and ramucirumab), and BRAF kinase (encorafenib). This coincided with an improved understanding of the biologic heterogeneity of CRC and the relationship between genomic alterations within the tumor and response to targeted therapies.

The first predictive genomic biomarker to be established as part of standard of care testing for patients with mCRC was the *KRAS* gene, which if mutated at specific codons, negated the benefit from anti-EGFR agents.^5^ A Canadian guidance document was published in 2011, which outlined recommendations for *KRAS* testing in mCRC^6^; however, other genomic biomarkers have since become important to inform the exclusion or inclusion of targeted agents in a patient’s treatment regimen. Furthermore, there is now an established role for immunotherapy checkpoint inhibitors (pembrolizumab, nivolumab, and ipilimumab) in biomarker-defined populations of mCRC.

Clinical trials in mCRC continue to take a biomarker-driven approach, with many new predictive biomarkers linked to pre-existing and novel therapies on the cusp of being clinically relevant. With no national guidelines reflecting current biomarker requirements in mCRC, this guideline was developed by a pan-Canadian advisory group to provide contemporary, evidence-based recommendations on the minimum acceptable standards for tumor biomarker testing in mCRC, and to describe emerging biomarkers for consideration.

## Guideline development

A pan-Canadian advisory group of medical oncologists and pathologists specializing in CRC was formed to develop the practice guideline. Consensus was reached on guideline methods and recommendation statements through two virtual meetings. Grading strength of recommendations was based on the GRADE system.^7^ (Table 1)

**Table 1.** Grading strength of recommendations based on GRADE system.^7^

Designation	Description	Rationale
Strong recommendation	Recommend for or against a particular molecular testing practice for colorectal cancer (can include must or should)	Supported by convincing or adequate strength of evidence, high or intermediate quality of evidence, and clear benefit that outweighs any harms
Recommendation	Recommend for or against a particular molecular testing practice for colorectal cancer (can include should or may)	Some limitations in strength of evidence (adequate or inadequate) and quality of evidence (intermediate or low), balance of benefits and harms, values, or costs, but panel concludes that there is sufficient evidence and/or benefit to inform a recommendation
Expert consensus opinion	Recommend for or against a particular molecular testing practice for colorectal cancer (can include should or may)	Serious limitations in strength of evidence (inadequate or insufficient), quality of evidence (intermediate or low), balance of benefits and harms, values, or costs, but panel consensus is that a statement is necessary
No recommendation	No recommendation for or against a particular molecular testing practice for colorectal cancer	Insufficient evidence or agreement of the balance of benefits and harms, values, or costs to provide a recommendation

The guideline development and literature search were focused on answering the following questions:

1. What tumor biomarkers are important to inform treatment selection in mCRC?
2. What tumor biomarkers have emerging actionability in mCRC?
3. What are the optimal methods for performing tumor biomarker testing in mCRC?
4. When should tumor biomarker testing be performed?

The literature search was conducted in two steps. First, international guidelines on biomarker testing and treatment for CRC were identified through an internet search of international health organizations. Since the last guideline from the American Society for Clinical Pathology, College of American Pathologists, Association for Molecular Pathology, and American Society of Clinical Oncology (ASCO) was published in February 2017 and included a systematic literature review at a publication cut-off date of February 2015,^8^ references from this publication were used to support guideline statements. The second step involved a literature search in MEDLINE using the OvidSP database, with publication cut-off dates between 1 February 2015 and 1 February 2022. Literature search included the terms ‘colorectal neoplasms’, ‘molecular targeted therapy’ or ‘antineoplastic agents’, and ‘biomarkers’. The search was filtered to include practice guidelines, consensus documents, systematic reviews, meta-analyses, randomized controlled trials, comparative studies, reviews, and evaluation studies. In addition to journal articles, the search identified meeting abstracts from ASCO, ASCO-Gastrointestinal Cancers Symposium, and European Society for Medical Oncology. Reference lists from identified publications were also scanned for additional relevant reports.

## Minimum biomarker testing standards in mCRC

**Figure 1.**
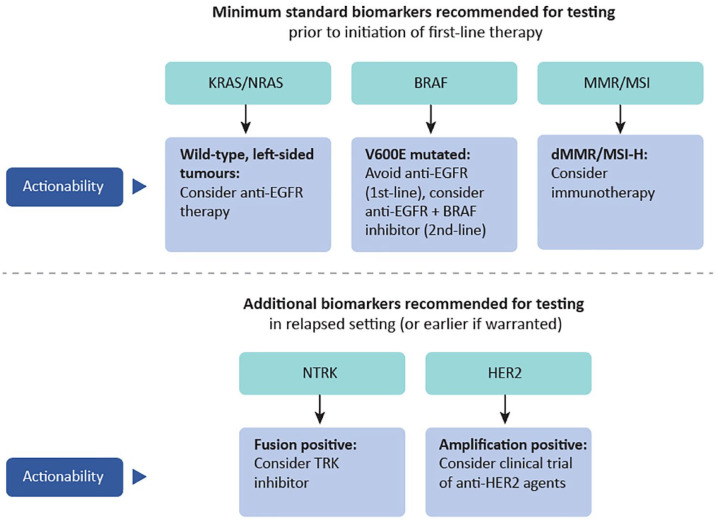
Summary of recommendations for testing of predictive tumor biomarkers in metastatic colorectal cancer. dMMR/MSI-H, mismatch repair deficient/microsatellite instability high.

This section states the minimum biomarker testing required across all Canadian jurisdictions for patients with CRC prior to initial treatment in the metastatic setting (Figure 1). Recommendations for assessment of these biomarkers are based on adequate evidence demonstrating clinical actionability, meaning the status of the biomarker is needed to inform likely response, benefit, and/or access to Health Canada-approved therapies (Table 2).

**Table 2.** Summary of recommendations and grading for tumor biomarker testing in metastatic colorectal cancer.

Statements	Grading
Minimum standard of care for tumor biomarker testing	
All patients with mCRC must have their tumor samples analyzed for: 1. Mutations in *KRAS* and *NRAS* genes (extended *RAS*), including at minimum codons 12 and 13 of exon 2, 59 and 61 of exon 3, and 117 and 146 of exon 4, to inform treatment decisions regarding anti-EGFR monoclonal antibodies: • In combination with chemotherapy in the first-line setting for left-sided primaries • In later lines of therapy 2. Mutations in *BRAF* V600: • For prognostic assessment • To inform treatment planning with anti-EGFR therapy • To inform treatment planning with combination *BRAF* inhibitor and anti-EGFR therapy in the second-line and beyond setting 3. MMR/MSI (if not previously assessed) • To inform treatment decisions regarding immune checkpoint inhibitors • To determine need for genetic testing for Lynch syndrome	Strong recommendation
Extended biomarker testing options	
4. Testing for *NTRK* gene fusions should be considered in patients with previously treated mCRC: • To inform eligibility for treatment with TRK inhibitors when no satisfactory treatment options are available	Recommendation
5. Testing for *HER2* gene copy number variations may be considered in patients with previously treated mCRC: • To inform eligibility for treatment with HER2-targeted therapy	Expert clinical opinion
6. Broad molecular testing, including but not limited to NTRK, HER2, and TMB, may be considered to inform eligibility for clinical trials in patients with refractory mCRC	Expert clinical opinion
7. There is insufficient evidence to support routine testing of TMB to inform treatment decisions with immune checkpoint inhibitors	No recommendation
Biomarker testing methodologies and reporting	
8. Biomarker testing for mCRC must be validated in accordance with best laboratory practices and be performed by an accredited laboratory that conforms to quality guidelines and routinely participates in proficiency testing, such as that offered by the College of American Pathologists	Strong recommendation
9. Biomarker testing results should be reported to the medical oncologist by the time of first consultation to inform first-line treatment decisions	Strong recommendation
10. Biomarker testing reports should conform to existing guidelines (American College of Medical Genetics, College of American Pathologists, Canadian College of Medical Geneticists), be understandable to medical oncologists, and should include description of testing method, sample adequacy, specific alteration detected with classification, and interpretation of results	Strong recommendation
11. Metastatic, recurrent, or primary CRC tissue are all acceptable specimens for IHC or molecular testing in mCRC; however, a new biopsy may be considered if the only available sample for testing is an FFPE tissue block older than 5 years from the primary diagnosis	Recommendation
12. Testing methods must be validated for FFPE. Testing on additional materials such as alcohol-fixed specimens may be performed but should be validated according to local practices	Recommendation
13. Multi-gene NGS panel testing should be considered to optimize turnaround time, utilization of tissue specimen, detection of actionable biomarkers, and to keep pace with evolving biomarker standards	Recommendation
14. When multi-gene panels are used, identification of alterations in genes outside of the minimum standard recommendations for mCRC should be reported to the medical oncologist	Expert clinical opinion

CRC, colorectal cancer; EGFR, epidermal growth factor receptor; FFPE, formalin-fixed, paraffin-embedded; HER2, human epidermal growth factor receptor 2; mCRC, metastatic colorectal cancer; MMR, mismatch repair; MSI, microsatellite instability; NGS, next-generation sequencing; NTRK, neurotrophic tyrosine receptor kinase; TMB, tumour mutational burden; TRK, tropomyosin receptor kinases.

### Extended RAS testing (including KRAS and NRAS)

Analysis of *KRAS* and *NRAS* mutation status is well-established as standard of care, with all international guidelines reviewed in the literature search recommending mutation testing for these genes (Table 3). These recommendations are based on the predictive value of *KRAS* and *NRAS* mutation status for the efficacy of cetuximab and panitumumab in patients with mCRC.

**Table 3.** Summary of international guidelines on biomarker testing and treatment for metastatic colorectal cancer.

Guideline	Date published/literature search date range	Biomarker summary			
		KRAS/NRAS	BRAF	MMR/MSI	Other
Canadian Expert Group consensus recommendations: KRAS testing in colorectal cancer. Aubin *et al.*^6^	August 2011 Reference range: 2007–2010	• Testing of codon 12/13 of KRAS as negative predictive biomarker for anti-EGFR therapy • Requested at start of second-line therapy for mCRC • No mention of NRAS	• BRAF is a negative prognostic factor, but not clear as predictive biomarker • Not recommended	• Not discussed	• Testing of PTEN, amphiregulin. epiregulin, PIK3CA not recommended
Molecular biomarkers for the evaluation of colorectal cancer: guideline from the ASCP, CAP, AMP, and ASCO Sepulveda *et al.*^8^	February 2017 Reference range: January 2008–February 2015	• KRAS and NRAS exons 2, 3, 4 recommended in patients considering anti-EGFR therapy as it is a negative predictive biomarker	• *BRAF* V600 mutation analysis recommended to be performed for prognostic stratification • *BRAF* V600 recommended to be performed in dMMR tumors with loss of MLH1 to evaluate Lynch syndrome risk • Insufficient evidence to recommend *BRAF* V600 mutational status as predictive biomarker for response to anti-EGFR therapy	• Recommend MMR status testing in CRC for identification of patients with high risk for Lynch syndrome and/or prognostic stratification	• Insufficient evidence to recommend PIK3CA, PTEN testing
ESMO consensus guidelines for the management of patients with metastatic colorectal cancer Van Cutsem *et al.* 2016^9^	July 2016 Reference range: 1991–2015	• KRAS and NRAS exons 2, 3, 4 recommended in all patients at time of diagnosis of mCRC as it is a negative predictive biomarker for anti-EGFR therapy	• *BRAF* mutation status is recommended to be assessed alongside RAS status (at diagnosis of mCRC) for prognostic assessment (and/or potential selection for clinical trials)	• Role of MSI as an independent prognostic biomarker in mCRC is unclear • MSI testing in mCRC can assist in genetic counseling • MSI strong predictive biomarker for benefit with immune checkpoint inhibitors	• Emerging biomarkers not recommended for routine patient management outside of a clinical trial setting: PIK3CA (exon 20), PTEN loss by IHC, amphiregulin, epiregulin, TGF-alpha, EGFR protein expression, amplification, copy number variations and mutations in ectodomain, *HER2* amplifications/mutations, HER3 and MET receptor overexpression
Pan-Asian adapted ESMO consensus guidelines for the management of patients with metastatic colorectal cancer: a JSMO–ESMO initiative endorsed by CSCO, KACO, MOS, SSO, and TOS Yoshino *et al.*^10^	January 2018 Reference range: 2005–2017	• Same as ESMO	• Same as ESMO except V600E mutation specified	• Same as ESMO except: • Specified both IHC testing for MMR and PCR testing for MSI could be used to inform genetic counseling • Specified tumor MMR testing has strong predictive value for use of checkpoint inhibitors	• Same as ESMO except detection of mutations in PIK3CA, exon 20 deemed optional
UK colorectal cancer NICE guideline (NG151)^11^	January 2020 Reference range: Up to 2018	• KRAS and NRAS testing is recommended as a negative predictive marker for anti-EGFR therapy	• BRAF V600E testing is recommended as a negative predictive marker for anti-EGFR therapy	• Recommended for all newly diagnosed patients with CRC	• N/A
Cancer Council Australia^12^	October 2017	• *KRAS* and *NRAS* mutation testing is recommended for patients with advanced CRC who are being considered for anti-EGFR therapy	• *BRAF* mutation testing is recommended in patients with advanced CRC as emerging evidence suggests poor response to anti-EGFR treatment	• dMMR testing is recommended in all CRC patients to identify Lynch syndrome	• N/A
NCCN guidelines version 3.2021 colon cancer^13^	September 2021 References up to 2020	• KRAS and NRAS exons 2, 3, 4 recommended in all patients with mCRC as it is a negative predictive biomarker for anti-EGFR therapy	• *BRAF* mutation genotyping (or IHC) is recommended in all patients with mCRC as *BRAF* V600E mutation makes response to anti-EGFR therapy highly unlikely unless given with a BRAF inhibitor	• MMR or MSI testing is recommended in all newly diagnosed patients with CRC	• HER2 testing via IHC, FISH, or NGS is noted as an option • NTRK fusion testing via multiple methods is noted as an option (although IHC positive requires confirmation) in patients with wild-type *KRAS*, *NRAS*, and *BRAF* and potentially who are dMMR/MSI-H

AMP, Association for Molecular Pathology; ASCO, American Society of Clinical Oncology; ASCP, American Society for Clinical Pathology; CAP, College of American Pathologists; CRC, colorectal cancer; CSCO, Chinese Society of Clinical Oncology; dMMR/MSI-H, mismatch repair deficiency/microsatellite instability high; EGFR, epidermal growth factor receptor; ESMO, European Society for Medical Oncology; FISH, fluorescence *in situ* hybridization; IHC, immunohistochemistry; JSMO, Japanese Society of Medical Oncology; KACO; Korean Association for Clinical Oncology; MMR, mismatch repair; MOS, Malaysian Oncological Society; MSI, microsatellite instability; NCCN, National Comprehensive Cancer Network; NGS, next-generation sequencing; NICE, The National Institute for Health and Care Excellence; PCR, polymerase chain reaction; SSO; Singapore Society of Oncology; TOS, Taiwan Oncology Society.

In the initial analyses of two phase III, randomized controlled trials, cetuximab or panitumumab in combination with best supportive care (BSC) demonstrated significantly prolonged progression-free survival (PFS) compared with BSC alone in unselected patients with relapsed mCRC.^14,15^ However, data reported from subsequent clinical studies of anti-EGFR monoclonal antibodies, including retrospective analyses of the aforementioned trials, demonstrated that benefit from these novel therapies was limited to *RAS* wild-type mCRC^5,16–26^ (Table 4). These findings have strong biologic plausibility given that RAS is an important molecule in the mitogen-activated protein kinase (MAPK) signaling pathway which functions downstream of EGFR. Indeed, in cellular models of CRC, mutations leading to activated KRAS proteins have demonstrated evasion of the MAPK signal-suppressing effects of EGFR inhibitors.^18^

**Table 4.** Clinical trials of anti-EGFR therapies with reported outcomes by *RAS* and *BRAF* mutation status.

Trial, design	Treatment arms	Population	Biomarkers evaluated	Number of patients	Results by biomarker status (HR or OR; [95% CI])
CO.17 Karapetis *et al.*^5^ Loree *et al.*^27^ Ph III	CET + BSC *versus* BSC	Advanced CRC no remaining SOC available *N* = 572	KRAS/NRAS codons 12, 13, 59, 61, 117, 146 *BRAF* V600E	KRASm_(ex 2)_ *n* = 164 KRASwt_(ex 2)_ *n* = 230 RASm_(ex 2,3,4):_ *n* = 213 BRAFm_(V600E)_ *n* = 15 RAS/BRAFwt *n* = 97	**KRASwt (ex 2)** PFS: HR_(CET + BSC *versus* BSC)_ = 0.40 [0.30–0.54]* OS: HR_(CET + BSC *versus* BSC)_ = 0.55; [0.41–0.74]* **KRASm (ex 2)** PFS: HR_(CET + BSC *versus* BSC)_ = 0.99; [0.73–1.35] OS: HR_(CET + BSC *versus* BSC)_ = 0.98; [0.70–1.37] **RASm (ex 2, 3, 4)** PFS: HR_(CET + BSC *versus* BSC)_ =1.04; [0.79–1.37] OS: HR_(CET + BSC *versus* BSC)_ = 0.91; [0.68–1.23] **BRAFm (V600E)** PFS: HR_(CET + BSC *versus* BSC)_ = 0.75; [0.26–2.19] OS: HR_(CET + BSC *versus* BSC)_ = 0.71; [0.22–2.27] **RAS/BRAFwt (ex 2, 3, 4 and V600E)** PFS: HR_(CET + BSC *versus* BSC)_ = 0.25; [0.15–0.41]* OS: HR_(CET + BSC *versus* BSC)_ = 0.51; [0.32–0.81]*
OPUS Bokemeyer *et al*.^24^ Ph II	CET + FOLFOX4 *versus* FOLFOX4	First-line EGFR-expressing mCRC *N* = 337	KRAS/NRAS codons 12, 13, 59, 61, 117, 146	KRASwt_(ex 2)_ *n* = 179 KRASm_(ex 2)_ *n* = 136 RASwt_(ex 2,3,4)_: *N* = 87 RASm_(ex 2,3,4)_: *n* = 167 RASm_(KRAS ex 2 wt)_: *n* = 31	**KRASwt (ex 2)** ORR: OR_(CET + FOLFOX4 *versus* FOLFOX4)_ = 2.55 [1.38–4.72] PFS: HR_(CET + FOLFOX4 *versus* FOLFOX4)_ = 0.57 [0.38–0.86]* **KRASm (ex 2)** ORR: OR_(CET + FOLFOX4 *versus* FOLFOX4)_ = 0.46 [0.23–0.92] PFS: HR_(CET + FOLFOX4 *versus* FOLFOX4)_ = 1.72 [1.10–2.68] **RASwt (ex 2, 3, 4)** ORR: OR_(CET + FOLFOX4 *versus* FOLFOX4)_ = 3.33 [1.36–8.17] PFS: HR_(CET + FOLFOX4 *versus* FOLFOX4)_ = 0.53 [0.27–1.04] **RASm (ex 2, 3, 4)** ORR: OR_(CET + FOLFOX4 *versus* FOLFOX4)_ = 0.58 [0.31–1.08] PFS: HR_(CET + FOLFOX4 *versus* FOLFOX4)_ = 1.54 [1.04–2.29] **RASm (KRAS ex 2** **wt)** ORR: OR_(CET + FOLFOX4 *versus* FOLFOX4)_ = 1.50 [0.34–6.53] PFS: HR_(CET + FOLFOX4 *versus* FOLFOX4)_ = 0.77 [0.28–2.08] ***Overall survival: No significant difference across any subgroups***
CRYSTAL Van Cutsem *et al.*^22^ Ph III	CET + FOLFIRI *versus* FOLFIRI	First-line EGFR-expressing mCRC *N* = 1198	KRAS/NRAS codons 12, 13, 59, 61, 117, 146	KRASwt_(ex 2)_ *n* = 666 RASwt_(ex 2,3,4):_ *N* = 367 RASm_(ex 2,3,4):_ *n* = 460 RASm_(KRAS ex 2 wt):_ *n* = 63	**KRASwt _(ex 2)_** PFS: HR_(CET + FOLFIRI *versus* FOLFIRI)_ = 0.70 [0.56–0.87]* OS: HR_(CET + FOLFIRI *versus* FOLFIRI)_ = 0.80 [0.67–0.95]* **RASwt (ex 2, 3, 4)** PFS: HR_(CET + FOLFIRI *versus* FOLFIRI)_ = 0.56 [0.41–0.76]* OS: HR_(CET + FOLFIRI *versus* FOLFIRI)_ = 0.69 [0.54–0.88]* **RASm (ex 2, 3, 4)** PFS: HR_(CET + FOLFIRI *versus* FOLFIRI)_ = 1.10 [0.85–1.42] OS: HR_(CET + FOLFIRI *versus* FOLFIRI)_ = 1.05 [0.86–1.28] **RASm (KRAS ex 2** **wt)** PFS: HR_(CET + FOLFIRI *versus* FOLFIRI)_ = 0.81 [0.39–1.67] OS: HR_(CET + FOLFIRI *versus* FOLFIRI)_ = 1.22 [0.69–2.16]
OPUS + CRYSTAL pooled analysis Bokemeyer *et al.*^28^	See above	See above	BRAF V600E	KRASwt_(ex 2)_/BRAFwt *n* = 730 KRASwt_(ex 2)_/BRAFm *n* = 70	**KRASwt (ex 2)/BRAFwt** PFS: HR_(CET + Chemo *versus* Chemo)_ = 0.64 [0.52–0.79]* OS: HR_(CET + Chemo *versus* Chemo)_ = 0.84 [0.71–1.00]* **KRASwt (ex 2)/BRAFm** PFS: HR_(CET +Chemo *versus* Chemo)_ = 0.67 [0.34–1.29] OS: HR_(CET + Chemo *versus* Chemo)_ = 0.62 [0.36–1.06]
COIN Maughan *et al.*^29^ Ph III	CET + FOLFOX *versus* FOLFOX (*versus* intermittent chemotherapy) *N* = 1630	First-line advanced CRC	KRAS codons 12, 13, 61 NRAS codons 12, 61 BRAF codons 594, 600	KRASwt *n* = 729 KRASm *n* = 565 KRASwt/BRAFm *n* = 102 RASwt/BRAFwt *n* = 581	**KRASwt (ex 2/codon 61)** PFS: HR_(CET + FOLFOX *versus* FOLFOX)_ = 0.96 [0.82–1.12] OS: HR_(CET + FOLFOX *versus* FOLFOX)_ = 1.04 [0.87–1.23] **KRASm (ex 2/codon 61)** OS: HR_(CET + FOLFOX *versus* FOLFOX)_ = 0.98 [0.81–1.17] **KRASwt (ex 2/codon 61)/BRAFm** OS: HR_(CET + FOLFOX *versus* FOLFOX)_ = 1.18 [0.76–1.81] **KRASwt (ex 2/codon 61)/BRAFwt** OS: HR_(CET + FOLFOX *versus* FOLFOX)_ = 1.02 [0.83–1.24]
NORDIC-VII Tveit *et al.*^30^ Guren *et al.*^31^ Ph III	FLOX *versus* CET + FLOX *versus* CET + intermittent FLOX *N* = 566	First-line mCRC	KRAS codons 12, 13, 61, 117, 146 NRAS codons 12, 13, 61, 146 BRAF codon 600	KRASwt_(ex 2)_ *n* = 303 KRASm_(ex 2)_ *n* = 195 RASwt/BRAFwt *n* = 192	**KRASwt (ex 2)** PFS: HR_(CET + FLOX *versus* FLOX)_ = 1.07 [0.79–1.45] OS: HR_(CET + FLOX *versus* FLOX)_ = 1.14 [0.80–1.61] **KRASm (ex 2)** PFS: HR_(CET + FLOX *versus* FLOX)_ = 0.71 [0.50–1.03] OS: HR_(CET + FLOX *versus* FLOX)_ = 1.03 [0.68–1.57] **RASwt (ex 2, 3, 4)/BRAFwt** PFS: HR_(CET + FLOX *versus* FLOX)_ = 1.06 [0.73–1.55] OS: HR_(CET + FLOX *versus* FLOX)_ = 1.07 [0.74–1.55]
CALGB/SWOG 80,405 Innocenti *et al*.^32^ Ph III	CET + Chemo *versus* BEV + Chemo *versus* CET + BEV + Chemo *N* = 2326 *N* = 843 with mutational analysis	First-line advanced, protocol amendment to restrict enrollment to KRAS ex 2 wt	KRAS codons 12, 13, 61, 117, 146 NRAS codons 12, 13, 61 BRAF codons 600, 601	RASwt *n* = 572 RASm *n* = 266 BRAFwt *n* = 743 BRAFm *n* = 100	**RASwt** PFS: HR_(BEV + Chemo *versus* CET + Chemo)_ = 0.90 [0.73–1.12] OS: HR_(BEV + Chemo *versus* CET + Chemo)_ = 0.91 [0.72–1.16] **RASm** PFS: HR_(BEV + Chemo *versus* CET + Chemo)_ = 0.77 [0.56–1.06] OS: HR_(BEV + Chemo *versus* CET + Chemo)_ = 1.07 [0.77–1.48] **BRAFwt** PFS: HR_(BEV + Chemo *versus* CET + Chemo)_ = 0.90 [0.74–1.09] OS: HR_(BEV +Chemo *versus* CET + Chemo)_ = 1.02 [0.83–1.25] **BRAFm** PFS: HR_(BEV + Chemo *versus* CET + Chemo)_ = 0.58 [0.33–1.02] OS: HR_(BEV + Chemo *versus* CET + Chemo)_ = 0.67 [0.37–1.20]
FIRE-3 Stintzing *et al.*^33^ Heinemann *et al.*^34^ Ph III	CET + FOLFIRI *versus* BEV + FOLFIRI *N* = 593	First-line mCRC, protocol amendment to restrict enrollment to KRAS ex 2 wt	KRAS/NRAS codons 12, 13, 59, 61, 117, 146 BRAF codons 466, 600	RASwt_(ex 2,3,4)_ *n* = 400 RASm_(ex 2,3,4)_ *N* = 188 BRAFm *n* = 48	**RASwt (ex 2, 3, 4)** PFS: HR_(CET + FOLFIRI *versus* BEV + FOLFIRI)_ = 0.96 [0.79–1.18] OS: HR_(CET + FOLFIRI *versus* BEV + FOLFIRI)_ = 0.76 [0.62–0.94]* **RASm (ex 2, 3, 4)** PFS: HR_(CET + FOLFIRI *versus* BEV + FOLFIRI)_ = 1.27 [0.94–1.70] OS: HR_(CET + FOLFIRI *versus* BEV + FOLFIRI)_ = 1.05 [0.78–1.42] **BRAFm** PFS: HR_(CET + FOLFIRI *versus* BEV + FOLFIRI)_ = 0.84 [0.47–1.51] OS: HR_(CET + FOLFIRI *versus* BEV + FOLFIRI)_ = 0.79 [0.43–1.46]
20020408 Amado *et al.*^16^ Patterson *et al.*^35^ Ph III	PAN + BSC *versus* BSC *N* = 463	Relapsed, EGFR-expressing mCRC	KRAS/NRAS codons 12, 13, 59, 61, 117, 146	KRASwt_(ex 2)_ *n* = 184 RASwt_(ex 2,3,4)_ *n* = NR RASm_(ex 2,3,4)_ *n* = NR	**KRASwt (ex 2)** PFS: HR_(PAN + BSC *versus* BSC)_ = 0.45 [0.34–0.59]* **RASwt (ex 2, 3, 4)** PFS: HR_(PAN + BSC *versus* BSC)_ = 0.38 [0.27–0.56]* **RASm (ex 2, 3, 4)** PFS: HR_(PAN + BSC *versus* BSC)_ = 0.98 [0.73–1.31]
20100007 Kim *et al*.^36^ Ph III	PAN + BSC *versus* BSC *N* = 377	Chemo-refractory KRAS exon 2 wt mCRC	KRAS/NRAS codons 12, 13, 59, 61, 117, 146 BRAF ex 15	KRASwt_(ex 2)_ *n* = 377 RASm_(KRAS ex 2 wt)_ *n* = 54 RASwt_(ex 2,3,4)_ *n* = 270 RASwt_(ex 2,3,4)_/BRAFwt *n* = 242 RASwt_(ex 2,3,4)_/BRAFm *n* = 20	**KRASwt (ex 2)** PFS: HR_(PAN + BSC *versus* BSC)_ = 0.54 [0.43–0.67]* OS: HR_(PAN + BSC *versus* BSC)_ = 0.74 [0.59–0.93]* **RASm (KRAS ex 2** **wt)** PFS: HR_(PAN +BSC *versus* BSC)_ = 1.03 [0.56–1.90] OS: HR_(PAN + BSC *versus* BSC)_ = 0.99 [0.49–2.00] **RASwt (ex 2, 3, 4)** PFS: HR_(PAN + BSC *versus* BSC)_ = 0.45 [0.35–0.59]* OS: HR_(PAN + BSC *versus* BSC)_ = 0.72 [0.55–0.94]* **BRAFwt/RASwt (ex 2, 3, 4)** PFS: HR_(PAN + BSC *versus* BSC)_ = 0.45 [0.34–0.60]* OS: HR_(PAN + BSC *versus* BSC)_ = 0.75 [0.57–0.99]* **BRAFm/RASwt (ex 2, 3, 4)** PFS: HR_(PAN + BSC *versus* BSC)_ = 0.28 [0.07–1.08] OS: HR_(PAN + BSC *versus* BSC)_ = 0.39 [0.10–1.51]
20050181 Peeters *et al.*^25^ Ph III	PAN + FOLFIRI *versus* FOLFIRI *N* = 1186	Previously treated mCRC	KRAS/NRAS codons 12, 13, 59, 61, 117, 146 BRAF codon 600	KRASwt_(ex 2)_ *n* = 597 KRASm_(ex 2)_ *n* = 486 RASwt_(ex 2,3,4)_ *n* = 421 RASm_(ex 2,3,4)_ *n* = 593 RASm_(KRAS ex 2 wt)_ *n* = 107 RASwt_(ex 2,3,4)_/BRAFwt *n* = 376 RASwt_(ex 2,3,4)_/BRAFm *n* = 45	KRASwt (ex 2) PFS: HR_(PAN + FOLFIRI *versus* FOLFIRI)_ = 0.73 [0.59–0.90]* OS: HR_(PAN + FOLFIRI *versus* FOLFIRI)_ = 0.85 [0.70–1.04] **KRASm (ex 2)** PFS: HR_(PAN + FOLFIRI *versus* FOLFIRI)_ = 0.85 [0.68–1.06] OS: HR_(PAN + FOLFIRI *versus* FOLFIRI)_ = 0.94 [0.76–1.15] **RASwt (ex 2, 3, 4)** PFS: HR_(PAN + FOLFIRI *versus* FOLFIRI)_ = 0.70 [0.54–0.91]* OS: HR_(PAN + FOLFIRI *versus* FOLFIRI)_ = 0.81 [0.63–1.03] **RASm (ex 2, 3, 4)** PFS: HR_(PAN + FOLFIRI *versus* FOLFIRI)_ = 0.86 [0.70–1.05] OS: HR_(PAN + FOLFIRI *versus* FOLFIRI)_ = 0.91 [0.76–1.10] **RASm (KRAS ex 2** **wt)** PFS: HR_(PAN + FOLFIRI *versus* FOLFIRI)_ = 0.89 [0.56–1.42] OS: HR_(PAN + FOLFIRI *versus* FOLFIRI)_ = 0.83 [0.53–1.29] **BRAFwt/RAS wt (ex 2, 3, 4)** PFS: HR_(PAN + FOLFIRI *versus* FOLFIRI)_ = 0.68 [0.51–0.90]* OS: HR_(PAN + FOLFIRI *versus* FOLFIRI)_ = 0.83 [0.64–1.07] **BRAFm/RAS wt (ex 2, 3, 4)** PFS: HR_(PAN + FOLFIRI *versus* FOLFIRI)_ = 0.69 [0.32–1.49] OS: HR_(PAN + FOLFIRI *versus* FOLFIRI)_ = 0.64 [0.32–1.28]
PICCOLO Seymour *et al.*^37^ Ph III	PAN + IRI *versus* IRI *versus* (IRI + ciclosporin) *N* = 1198	Advanced, chemoresistant, relapsed CRC, protocol amendment to restrict enrollment in PAN + IRI arm for KRAS wt only	KRAS/NRAS codons 12, 13, 59, 61, 117, 146 BRAF codon 600 PIK3CA ex 9, 20	wt_(RAS, BRAF, PIK3CA)_ *n* = 323 m_(RAS, BRAF, PIK3CA)_ *n* = 137 BRAFm *n* = 68	**Wild-type (*RAS, BRAF, PIK3CA*)** PFS: HR_(PAN + IRI *versus* IRI)_ = 0.68 [0.53–0.86]* OS: HR_(PAN + IRI *versus* IRI)_ = 0.92 [0.73–1.16] **Mutated (*RAS, BRAF, PIK3CA*)** PFS: HR_(PAN ++ IRI *versus* IRI)_ = 1.20 [0.83–1.74] OS: HR_(PAN + IRI *versus* IRI)_ = 1.64 [1.14–2.34] **BRAFm** PFS: HR_(PAN + IRI *versus* IRI)_ = 1.40 [0.82–2.39] OS: HR_(PAN + IRI *versus* IRI)_ = 1.84 [1.10–3.08]
PRIME Douillard *et al.*^26^ Ph III	PAN + FOLFOX4 *versus* FOLFOX4 *N* = 1183	First-line mCRC	KRAS/NRAS codons 12, 13, 59a, 61, 117, 146	KRASwt_(ex 2)_ *n* = 656 KRASm_(ex 2)_ *n* = 440 RASwt_(ex 2,3,4)_ *N* = 512 RASm_(ex 2,3,4)_ *n* = 548 RASm_(KRAS ex 2 wt)_ *n* = 108	**KRASwt (ex 2)** PFS: HR_(PAN + FOLFOX4 *versus* FOLFOX4)_ = 0.80 [0.66–0.97]* OS: HR_(PAN + FOLFOX4 *versus* FOLFOX4)_ = 0.83 [0.70–0.98]* **KRASm (ex 2)** PFS: HR_(PAN + FOLFOX4 *versus* FOLFOX4)_ = 1.29 [1.04–1.62] OS: HR_(PAN + FOLFOX4 *versus* FOLFOX4)_ = 1.16 [0.94–1.41] **RASwt (ex 2, 3, 4)** PFS: HR_(PAN + FOLFOX4 *versus* FOLFOX4)_ = 0.72 [0.58–0.90]* OS: HR_(PAN + FOLFOX4 *versus* FOLFOX4)_ = 0.77 [0.64–0.94]* **RASm (ex 2, 3, 4)** PFS: HR_(PAN + FOLFOX4 *versus* FOLFOX4)_ = 1.31 [1.07–1.60] OS: HR_(PAN + FOLFOX4 *versus* FOLFOX4)_ = 1.21 [1.01–1.45] **RASm (KRAS ex 2** **wt)** PFS: HR_(PAN + FOLFOX4 *versus* FOLFOX4)_ = 1.28 [0.79–2.07] OS: HR_(PAN + FOLFOX4 *versus* FOLFOX4)_ = 1.39 [0.91–2.13]
PEAK Rivera *et al.*^38^ Ph II	PAN + mFOLFOX6 *versus* BEV + mFOLFOX6 *N* = 285	First-line, KRAS ex 2 wt mCRC	KRAS/NRAS codons 12, 13, 59, 61, 117, 146 BRAF codon 600	RASwt_(ex 2,3,4)_ *n* = 170 BRAFwt *n* = 156	**RASwt (ex 2, 3, 4)** PFS: HR_(PAN + FOLFOX6 *versus* BEV + FOLFOX6)_ = 0.68 [0.48–0.96]* OS: HR_(PAN + FOLFOX6 *versus* BEV + FOLFOX6)_ = 0.76 [0.53–1.11] **RASwt (ex 2, 3, 4)/BRAF wt** PFS: HR_(PAN +FOLFOX6 *versus* BEV + FOLFOX6)_ = 0.61 [0.42–0.88]* OS: HR_(PAN + FOLFOX6 *versus* BEV + FOLFOX6)_ = 0.70 [0.48–1.04]
PARADIGM Yoshino *et al.*^39^ Ph III	PAN + mFOLFOX6 *versus* BEV + mFOLFOX6 *N* = 802	First-line, KRAS/NRAS ex 2/3/4 wt mCRC	KRAS/NRAS codons 12, 13, 59, 61, 117, 146	RASwt_(ex 2,3,4)_ *n* = 802	**RASwt (ex 2, 3, 4)** PFS: HR_(PAN + FOLFOX6 *versus* BEV + FOLFOX6)_ = 1.01 [0.87–1.18] OS: HR_(PAN + FOLFOX6 *versus* BEV + FOLFOX6)_ = 0.84 [0.72–0.98]*

* Denotes statistically significant reduction in risk for anti-EGFR therapy arm

BEV, bevacizumab; BSC, best supportive care; CET, cetuximab; Chemo, chemotherapy; CI, confidence interval; CRC, colorectal cancer; EGFR, epidermal growth factor receptor; ex, exon; FLOX, bolus fluorouracil/folinic acid and oxaliplatin; FOLFIRI, 5-fluorouracil, leucovorin, irinotecan; FOLFOX, 5-fluorouracil, leucovorin, oxaliplatin; HR, hazard ratio; IRI, irinotecan; m, mutated; mCRC, metastatic colorectal cancer; NR, not reported; OR, odds ratio; OS, overall survival; PAN, panitumumab; PFS, progression-free survival; SOC, standard of care; wt, wild-type.

Missense mutations in *KRAS* and *NRAS* genes have been reported in approximately 50 and 5% of advanced CRCs, respectively, with the majority of mutations occurring in codons 12 and 13 within exon 2 of *KRAS*.^40^ Because of this high mutational frequency, most trial analyses initially evaluated efficacy outcomes based only on *KRAS* codon 12 and 13 mutation status. However, an exploratory analysis of the PRIME trial showed that missense mutations in exons 3 and 4 of *KRAS* and exons 2, 3, and 4 of *NRAS* occurred in a combined 17% of patients and were also indicators of inferior PFS and OS in patients with mCRC receiving panitumumab plus FOLFOX.^26^ Other *post hoc* analyses of clinical trials and meta-analyses have confirmed these trends showing mutations in *KRAS* and *NRAS* in codons 12 and 13 of exon 2, 59 and 61 of exon 3, and 117 and 146 of exon 4 are negative predictors for response to EGFR targeted therapies^41,42^ (Table 4).

Location of primary tumor has also been shown to impact prognosis and response to anti-EGFR therapy, with retrospective analyses from the Intergroup 80405, CRYSTAL, FIRE-3, PEAK, PRIME, and PARADIGM trials showing that patients with left-sided tumors, but not those with right-sided tumors, benefited from the addition of anti-EGFR therapy to their treatment (Yoshino, and , 2021).^43–45^ Incorporation of cetuximab or panitumumab with FOLFIRI or FOLFOX are now standard of care first-line treatment options in Canada for patients with mCRC who have left-sided primary tumors and are *RAS* wild-type.^46,47^ Some clinicians may choose to avoid upfront anti-EGFR therapy in combination with chemotherapy in patients with resectable liver metastases, based on the New EPOC data, but this remains a controversial area.^48^

In addition to serving as a biomarker to exclude patients from receiving anti-EGFR therapy, therapies targeting the *KRAS* G12C mutation, which occur in 3–4% of CRCs,^49^ are under investigation. This includes the small molecule inhibitors sotorasib and adagrasib, which bind specifically to the inactive GDP-bound state of *KRAS* G12C mutant proteins. Early phase trials have reported overall response rates (ORRs) of 7 and 22%, for these agents as monotherapy in relapsed mCRC, respectively.^50,51^ The phase III KRYSTAL-10 study evaluating adagrasib plus cetuximab *versus* chemotherapy in patients with relapsed advanced CRC and *KRAS* G12C mutations is ongoing.^52^

### *BRAF* V600 testing

The BRAF protein is a serine/threonine protein kinase functioning downstream of RAS in the MAPK signaling pathway. Activating V600 mutations in the *BRAF* gene are considered mutually exclusive with *RAS* mutations and occur in approximately 10% of mCRC cases. *BRAF* V600E mutations tend to be enriched in right-sided tumors and tumors with high microsatellite instability (MSI-H).^53–55^ Compared with *BRAF* wild-type CRC, tumors harboring *BRAF* V600E mutations have been independently correlated with worse survival and rapid disease progression following first-line chemotherapy.^54–56^

The perceived value of *BRAF* mutation analysis has evolved over the last 15 years. Guidelines from European Society for Medical Oncology (ESMO) and ASCO published in July 2016 and February 2017, respectively, acknowledge the prognostic value of *BRAF* V600E mutations; however, they stated that there was insufficient evidence to conclude that patients with *BRAF*-mutated CRC do not benefit from anti-EGFR therapies, and therefore should not be used as a predictive biomarker^8,9^ (Table 3). This statement is based on the difficulty in discerning the predictive value of *BRAF* V600E mutations due to low mutational prevalence and association with other poor prognostic features. In addition, a meta-analysis by Rowland *et al.* pooling data from eight RCTs, showed a lack of PFS benefit with anti-EGFR therapies in *BRAF*-mutated patients [hazard ratio (HR) 0.86 (95% CI: 0.61–1.21)] and a significant PFS improvement in *BRAF* wild-type patients [HR 0.62 (95% CI: 0.50–0.77)]; however, the interaction test to detect a difference was just outside the threshold of significance (*p* = 0.07).^57^ Other groups have argued that although not statistically significant, the *p*-value of the interaction test is clinically relevant,^58^ and the body of evidence to support the lack of benefit to anti-EGFR therapies in *BRAF*-mutated mCRC, including a series of individual studies and meta-analyses, is equivalent, if not superior, to that of *RAS* mutations outside of *KRAS* exon 2^59^ (Table 4). Assessment of *BRAF* mutation status is recommended in guidelines published by The National Institute for Health and Care Excellence (NICE), National Comprehensive Cancer Network (NCCN), and Cancer Council Australia to select patients most likely to respond to anti-EGFR therapies (Table 3).

*BRAF* mutation status is additionally recommended to select patients for treatment with BRAF inhibitors. Although BRAF inhibitor monotherapy is effective in patients with melanoma and *BRAF* V600E mutations, it has produced low response rates in *BRAF* V600E-mutated CRCs.^60^ Evidence from preclinical studies suggest that this lack of response is caused by feedback reactivation of EGFR and subsequent initiation of downstream signaling.^61,62^ For this reason, combination therapies targeting multiple points along the MAPK pathway have been investigated in *BRAF* V600-mutated CRC. The phase II SWOGS1406 study in relapsed mCRC demonstrated that the addition of the BRAF inhibitor vemurafenib to irinotecan and cetuximab resulted in improved PFS, ORR, and disease control rate for patients with *BRAF* V600E mutations compared with cetuximab and irinotecan alone.^63^ A phase I study of the BRAF and MEK inhibitors, dabrafenib and trametinib also demonstrated activity in patients with *BRAF* V600E-mutated mCRC.^64^ Results from the pivotal phase III BEACON study led to the Health Canada approval of encorafenib (BRAF inhibitor) plus cetuximab for patients with previously-treated *BRAF* V600E-mutated mCRC. This study examined encorafenib in combination with cetuximab, with or without the MEK inhibitor binimetinib *versus* investigator’s choice of irinotecan or FOLFIRI plus cetuximab.^65^ At a median follow-up of 12.8 months, both the doublet and triplet encorafenib regimens demonstrated superior OS compared to the control arm (median OS 9.3 months for both arms *versus* 5.9 months for control; HR 0.60, 95% CI: 0.47–0.75 for triplet *versus* control and HR 0.61, 95% CI: 0.48–0.77 for doublet *versus* control).

Encorafenib combination therapies are also being investigated in the first-line setting for patients with *BRAF* V600E-mutated mCRC. This includes the phase II ANCHOR study, which met its primary endpoint with an ORR of 47.8% for encorafenib, binimetinib, and cetuximab and a median PFS and OS of 5.8 and 17.2 months, respectively.^66^ The phase III BREAKWATER trial evaluating encorafenib plus cetuximab with or without chemotherapy for first-line treatment of *BRAF* V600E-mutated mCRC is ongoing.^67^

### Mismatch repair deficiency/microsatellite instability testing

Alterations in genes responsible for DNA mismatch repair (MMR) lead to changes in the length of short, tandemly repeated DNA motifs – a genomic phenotype termed microsatellite instability (MSI). Less than one-third of CRC cases with MMR deficiency (dMMR)/MSI-H have germline mutations in *MMR* genes (*MLH1*, *MSH2*, *PMS2*, and *MSH6*) which are linked to an inherited condition of cancer susceptibility called Lynch syndrome.^8^ International guidelines recommend testing for MMR status in all patients with CRC to inform the need for cascade testing of family members and subsequent risk-reduction strategies in those identified with Lynch syndrome.(Table 3)

The frequency of dMMR/MSI and its significance in the management of CRC varies by disease stage. It occurs in approximately 20, 12, and 5% of patients with stage II, III, and IV CRC, respectively.^68^ In stage II–III CRC, dMMR/MSI-H strongly correlates with an improved prognosis compared with MMR proficient/microsatellite stable (pMMR/MSS) tumors and is a predictor for lack of benefit from fluoropyrimidine monotherapy in stage II patients.^69,70^ Conversely, dMMR/MSI-H appears to be associated with worse prognosis in patients with mCRC.^71–75^ This finding may be related to the enrichment of *BRAF* V600 mutations in patients with sporadic dMMR/MSI mCRC.^74,76^

International guidelines have acknowledged the emerging value of MMR and MSI testing to predict response to immune checkpoint inhibitors. In early phase clinical trials, the anti-programmed death-1 (PD-1) receptor antibody, pembrolizumab, showed activity in patients with dMMR/MSI-H mCRC, with ORRs between 33 and 53%.^77–79^ Results from the pivotal phase III KEYNOTE-177 trial led to the Health Canada approval of pembrolizumab monotherapy as first-line treatment for patients with dMMR/MSI-H mCRC. In this trial, pembrolizumab treatment resulted in significantly prolonged PFS compared with the control arm of FOLFOX or FOLFIRI with or without bevacizumab or cetuximab (median, 16.5 *versus* 8.2 months; HR 0.60, 95% CI: 0.45–0.80; *p* = 0.0002).^80^ At a median follow-up of 44 months, there was also a trend for prolonged OS with pembrolizumab (median not reached *versus* 36.7 months; HR 0.74, 95% CI: 0.53–1.03; *p* = 0.0359); however, statistical significance was likely not met due to the high rate of patients receiving subsequent immune checkpoint inhibitors (60%).^81^

The anti-PD-1 antibody nivolumab also has conditional approval from Health Canada, in combination with the anti-cytotoxic T-lymphocyte-associated antigen 4 agent ipilimumab, for patients with dMMR/MSI-H mCRC after prior fluoropyrimidine-based therapy in combination with oxaliplatin or irinotecan. This was based on results from the multi-cohort, phase II CHECKMATE 142 study, where patients treated with nivolumab and ipilimumab achieved an ORR of 55% and a disease control rate for ⩾12 weeks of 80%.^82^ In another cohort of patients with previously untreated mCRC, nivolumab plus ipilimumab, achieved an ORR and disease control rate of 69 and 84%, respectively. At a median follow-up of 29.0 months, median PFS and OS were not reached.^83^

## Extended biomarker testing options

In addition to the minimum required biomarkers for testing in mCRC, the panel has agreed that the following biomarkers could be considered during later lines of therapy. These actionable biomarkers are required either to access current Health Canada-approved therapies or to confirm eligibility for ongoing clinical trials. Testing for these biomarkers may be considered earlier in the metastatic setting if a patient is not a good candidate for traditional chemotherapy, and they may be incorporated into initial testing when multi-gene next-generation sequencing (NGS) panels are used. It is important to acknowledge that publicly funded access to biomarker-linked therapies may vary across jurisdictions, which should be discussed with the patient.

### NTRK testing

Neurotrophic tyrosine receptor kinase (*NTRK*) genes encode a family of transmembrane-receptor proteins, called tropomyosin receptor kinases (TRKs), which are involved in neural development.^84^ Translocations in *NTRK1*, *NTRK2*, and *NTRK3* genes (encoding TRKA, TRKB, and TRKC proteins) have gained enormous interest since the first gene fusion was detected in 1982, in a colorectal adenocarcinoma cell line.^85^ Since then, over 80 different gene fusion partners have been identified across many tumor types.^84^ These fusions typically involve the portion of an *NTRK* gene, which encodes for the tyrosine kinase domain joined with portions of genes that encode for dimerization motifs.^84^ In this way, TRK proteins become constitutively activated and contribute to cancer pathogenesis through aberrant signaling of the MAPK and PI3K pathways.

*NTRK* gene fusions are now clinically actionable in any cancer type based on results from clinical trials investigating the TRK inhibitors larotrectinib and entrectinib. A pooled analysis of three trials evaluating larotrectinib monotherapy in 153 adult and pediatric patients with refractory cancers of various tumor histologies demonstrated an ORR of 79% and CR rate of 16%.^86^ Responses were durable, leading to a median PFS of 28.3 months. Entrectinib, which targets TRK proteins, as well as c-ROS oncogene1 (*ROS*) and anaplastic lymphoma kinase (*ALK*), was studied in the STARTRK-1, STARTRK-2, and ALKA372-001 trials. A pooled analysis of these trials, including 54 adult patients with refractory malignancies, demonstrated an ORR of 57%, CR rate of 7%, and median PFS of 11.2 months.^87^ Although subgroups of patients with CRC in these trials were small, response rates appeared lower than in the overall populations, with four of eight patients (50%) responding to larotrectinib and one of four patients (25%) responding to entrectinib. Additional studies are needed to better understand potential resistance mechanisms and whether patients with CRC benefit less from TRK inhibitors compared to patients with other tumor types.^88^

Several methods can be used to detect *NTRK* gene fusions, including immunohistochemistry (IHC), fluorescence *in situ* hybridization (FISH), reverse transcription polymerase chain reaction, and NGS. There are also multiple assays available using each method, with different advantages and limitations for each. The optimal assay for testing *NTRK* gene fusions should thus be decided at each institution based on the testing parameters and outputs.^84,89,90^ The ongoing CANTRK Ring study, which aims to harmonize and standardize Canadian molecular pathology laboratory approaches to NTRK testing, will also provide insight on optimal testing methods.^91^

Given the low incidence of *NTRK* gene fusions in CRC (approximately 0.2%),^92,93^ methods to improve cost-effectiveness of testing should be considered. A Canadian consensus statement on biomarker testing and treatment of patients with cancers harboring NTRK fusions proposes that costs may be reduced by first screening patients for TRK protein expression via IHC, followed by confirmation of *NTRK* gene fusion using NGS.^94^ Costs may further be reduced by identifying subgroups of patients where *NTRK* gene fusions are enriched. Since *NTRK* gene fusions are typically mutually exclusive to other oncogenic drivers such as *RAS* and *BRAF* mutations,^92,95^ and across multiple clinical trials, 76–89% of patients with TRK-fusion positive CRC were also dMMR/MSI-H,^92,95–98^ *RAS* and *BRAF* wild-type, dMMR/MSI-H CRCs may be an ideal target population for routine *NTRK* testing. The NCCN guidelines recommend limiting *NTRK* testing to this subpopulation, which account for less than 5% of patients with mCRC.^94^ Testing for NTRK fusions prior to first-line treatment may also be considered in select patients who are not good candidates for cytotoxic chemotherapy.

### HER2 testing

The *ERBB2* gene (herein referred to as *HER2*) encodes for the ErbB2 (HER2) protein, which is part of a family of receptor tyrosine kinases, including EGFR, ErbB3, and ErbB4. Heterodimerization of any two ErbB family proteins initiates the activation of MAPK, PI3K, Protein Kinase C, and Stress Activated Protein Kinase pathways.^99^ Around 2–5% of CRCs harbor *HER2* gene amplifications,^100–102^ and their occurrence is enriched in *RAS* and *BRAF* wild-type CRCs.^103^ *HER2* amplifications do not appear to be correlated with worse survival in CRC^104^; however, evidence from small, retrospective studies show that *HER2* amplifications are correlated with poorer response to anti-EGFR therapies.^105–108^ This supports the value of *HER2* amplification testing to inform treatment with anti-EGFR therapies.

While therapies targeting HER2 have become standard of care for the treatment of breast and gastroesophageal cancers with HER2 overexpression/gene amplifications, similar therapies are emerging for treating this subpopulation of patients with mCRC. The phase II HERACLES trial evaluated trastuzumab (an anti-HER2 antibody) and lapatinib (a small molecule inhibitor of HER2 and EGFR) in 35 patients with HER2-positive refractory mCRC, as determined by IHC and FISH.^109^ In the 32 patients evaluable for response, this dual HER2-targeted treatment produced an ORR of 28%, a CR rate of 3% (one patient), and 41% had stable disease. Median PFS was 4.7 months (95% CI: 3.7–6.1), and median OS was 10.0 months (95% CI: 7.9–15.8). Of note, central nervous system (CNS) metastasis occurred in 19% of patients, a high frequency, which mirrors disease progression outcomes with HER2-targeted therapies in breast and gastric cancers.^110^ Therefore, evidence of *HER2* amplification in mCRC should prompt vigilance in monitoring for CNS metastases, and presence of CNS metastases in CRC patients should prompt clinicians to consider testing for *HER2* amplification regardless of therapy line.^111,112^

Clinical trials evaluating other combinations of HER-targeted therapies in patients with *HER2*-amplified mCRC are ongoing, with early analyses demonstrating response rates between 25 and 55% (Table 5). Notably, the phase II DESTINY-CRC01 evaluated trastuzumab deruxtecan, an anti-HER2 antibody–drug conjugate, in 78 patients with previously treated, *RAS*-wild-type, HER2-expressing mCRC.^113^ Results reported for three cohorts based on HER2 expression level showed a 45% ORR for patients in cohort A [IHC 3+ or IHC 2+ and *in situ* hybridization (ISH) positive] and no confirmed response in either cohorts B or C (IHC 2+ and ISH negative or IHC 1+). In a subgroup analysis of cohort A, higher response rates were observed among patients with higher HER2 expression (ORR for IHC 3+ *versus* IHC 2+: 57.5 *versus* 7.7%).^118^ The NCCN guidelines for CRC recommend testing for *HER2* amplifications for patients with mCRC unless *RAS/BRAF* mutations have already been confirmed as *HER2* amplification is rare in this subgroup of patients.^102,105^

**Table 5.** Clinical trials evaluating HER2-targeted therapies in patients with *HER2*-amplified mCRC.

Trial name, Phase	Treatment arms	Study population	HER2 requirements	Outcomes
HERACLES^102,109^ Ph II	Trastuzumab + lapatinib	KRAS exon 2 wt HER2-positive Metastatic CRC Refractory to standard therapy *N* = 35	Tumors with 3+ HER2 score in >50% of cells by IHC or with 2+ HER2 score and a HER2:CEP17 ratio >2 in >50% of cells by FISH	ORR: 28% Median PFS: 4.7 months (95% CI: 3.7–6.1) Median OS: 10.0 months (95% CI: 7.9–15.8)
MOUNTAINEER^114,115^ Ph II	Trastuzumab + tucatinib Tucatinib monotherapy	RAS wt *HER2*-amplified Metastatic CRC Previously treated with 5FU, oxaliplatin, irinotecan, and an anti-VEGF antibody *N* = 22	*HER2* amplification detected by NGS, FISH, or IHC (3+ or 2+ and amplified by FISH)	ORR: 55% Median PFS: 6.2 months (95% CI: 3.5–NE) Median OS: 17.3 months (95% CI: 12.3–NE)
MyPathway^116^ Ph II basket study	Trastuzumab + pertuzumab	HER2-amplified Metastatic CRC Treatment refractory *N* = 57	*HER2* amplification detected by NGS, FISH, or IHC (3+ or 2+ and amplified by FISH)	ORR: 32% CR: 2% (one patient) Median PFS: 2.9 months (95% CI: 1.4–5.3)
TAPUR^117^ Ph II basket study	Trastuzumab + pertuzumab	HER2-positive Advanced CRC No standard treatment options *N* = 28	*HER2* amplification detected by NGS, FISH, or IHC and specific *HER2* mutations	DCR: 50% ORR: 25%
DESTINY-CRC01^113^ Ph II	Trastuzumab deruxtecan	RAS/BRAF wt HER2-positive Metastatic CRC ⩾2 prior therapies *N* = 78	Cohort A: IHC 3+ or IHC 2+ and FISH positive Cohort B: IHC 2+ and ISH negative Cohort C: IHC 1+	ORR: 45% in Cohort A No response in Cohorts B or C Median PFS: 6.9 months (95% CI: 4.1–NE)
DESTINY-CRC02 (NCT04744831) Ph II	Trastuzumab deruxtecan	HER2-positive Locally advanced, unresectable, or metastatic CRC Previously treated Projected *N* = 120	HER2 3+ by IHC or HER2 2+ and positive ISH	Primary endpoint: ORR by BICR

BICR, blinded independent central review; CI, confidence interval; CR, complete response; DCR, disease control rate; FISH, fluorescence *in situ* hybridization; 5FU, 5-fluorouracil; IHC, immunohistochemistry; NE, not evaluable; NGS, next-generation sequencing; ORR, overall response rate; OS, overall survival; PFS, progression-free survival; VEGF, vascular endothelial growth factor; wt, wild-type.

Several technologies can be used to test for *HER2* amplifications, although the optimal testing method is unclear. Many clinical trials in mCRC have followed the methods described in the HERACLES study, which define HER2 positivity as tumors with 3+ HER2 score in >50% of cells by IHC or with 2+ HER2 score and a HER2:CEP17 ratio >2 in >50% of cells by FISH.^102,113,114^ These are similar to the criteria for determining HER2 status in breast and gastroesophageal cancers except that the latter guidelines have a lower threshold for percentage of cells requiring positive staining (>10%).^119,120^ The TAPUR and MyPathway basket studies allow *HER2* detection by NGS, in addition to detection by IHC and/or FISH.^116,117^ Testing for *HER2* variations may be ideally evaluated within a multi-gene NGS panel; however, not all panels allow for detection of copy number variations and further clinical validation would be required.

### Tumor mutational burden testing

Tumor mutational burden (TMB) is a measure of the rate of somatic mutations occurring across all coding regions in a tumor genome. High TMB (TMB-H) leads to the production of tumor neoantigens, which increase the likelihood of stimulating an anti-tumor immune response. TMB has been assessed as a biomarker to predict response to immune checkpoint inhibitors. Since TMB is a continuous variable, thresholds for defining TMB-H vary among studies. In the phase II KEYNOTE-158 study, patients with a variety of solid tumors that were TMB-H, defined as 10 mutations/megabase (Mb) using the FoundationOne NGS assay, achieved an ORR of 29% with pembrolizumab treatment, compared to an ORR of 6% in the non-TMB-H cohort.^121^ Notably, patients with mCRC were not included as a cohort in this study. Based on these results, the U.S. Food and Drug Administration granted accelerated approval to pembrolizumab for the treatment of unresectable or metastatic solid tumors with TMB-H (⩾10 mutations/Mb), using the FoundationOne companion diagnostic assay. However, pembrolizumab has not been approved by Health Canada for this indication.

The frequency of TMB-H in CRC is approximately 3% and is strongly correlated with MSI-H status.^122^ In a study evaluating over 6000 CRC cases, 99.7% of MSI-H tumors were found to also have a TMB of ⩾12 mutations/Mb, whereas only 3% of pMMR/MSS cases were TMB-H.^122^ The ability of TMB-H to predict response to pembrolizumab in MSS mCRC remains unclear. The Targeted Agent and Profiling Utilization Registry (TAPUR) study assessed the efficacy of pembrolizumab in 27 patients with refractory MSS mCRC and TMB-H at a cut-off of ⩾9 mutations/Mb.^123^ This study found an ORR of only 11% and PFS of 9.3 weeks in patients with refractory mCRC receiving pembrolizumab monotherapy. Another study based in Japan found that 8 of 24 patients with pMMR/MSS CRC responded to a combination of regorafenib and nivolumab; however, no relationship between TMB-H and response was detected.^124^ In a study by the Canadian Cancer Trials Group, which randomized 180 patients with refractory mCRC to treatment with durvalumab and tremelimumab or BSC, patients with plasma TMB ⩾ 28 mutations/Mb had a greater OS benefit (HR 0.34; 90% CI: 0.18–0.63; *p* = 0.004) compared to the overall population (HR 0.72; 90% CI: 0.54–0.97; *p* = 0.07).^125^ However, in this same trial, the use of tissue TMB as a biomarker did not identify a group of patients with improved outcome following durvalumab and tremelimumab, and a cut point of 10 mutations/Mb did not result in improved outcomes (HR 0.54, 90% CI: 0.27–1.08, *p* = 0.14).^126^ This suggests that optimization and validation of different TMB thresholds for different tumor types may be needed.

### Other emerging predictive genomic alterations

Within the set of genes that are recommended to be assessed in mCRC, including *KRAS/NRAS* and *BRAF*, different types of genomic alterations that occur at a lower frequency are emerging as potential predictive biomarkers that require further validation. This includes *RAS* gene amplifications, which occur in 1–2% of patients with CRC and may be enriched in patients with a history of inflammatory bowel disease.^40,127,128^ Non-V600E *BRAF* missense mutations occur in up to 2% of mCRC cases and continue to be investigated as predictors of anti-EGFR therapy response.^129^ Some studies have reported different *BRAF* mutations having different impacts on response to anti-EGFR therapy, with one retrospective study showing reduced response in cases with mutations in codons 597 and 601 of *BRAF* compared to cases with mutations in codons 594 and 596.^130^ Another study did not observe responses to anti-EGFR therapies in any atypical *BRAF*-mutated patients with CRC; however, stable disease was achieved in 6 of 11 patients (50%).^131^ Genomic alterations in *ERRB* family genes other than *HER2* amplifications may also be predictors of response to anti-EGFR therapies but require validation. These include missense mutations or insertion/deletions with *HER2* and amplifications in ERRB3/HER3 or ERBB1/EGFR genes.^132,133^ Missense mutations within the *HER2* gene occur in approximately 3% of CRCs.^101^ Thus far, patients with mCRC harboring tumor *HER2* mutations have not responded to single-agent HER2 small molecule inhibitors in clinical trials^134^; however, this may be due to the varying sensitivities of different *HER2* mutations to anti-HER2 monotherapy.^135^ In addition, only clinical trials of anti-HER2 combination therapies, not monotherapy, have demonstrated efficacy in HER2-expressing mCRC.^136^ Dual HER2-targeted therapy has demonstrated anti-tumor activity in preclinical studies using xenograft models of *HER2*-mutated mCRC^137^ and anti-HER2 combination regimens continue to be evaluated in clinical trials for *HER2*-positive patients with mCRC (NCT05350917, NCT03457896, NCT04639219, and NCT04579380).

Mutations in the *PIK3CA* gene occur in 10–20% of patients with CRC and are commonly found in exon 9 (within the helical domain) and exon 20 (within the kinase domain). Given the role of PI3K in signal transduction downstream of EGFR, *PIK3CA* mutations have also been considered a contributor to the lack of response to anti-EGFR therapy observed in some *RAS* wild-type patients.^138^ Studies have reported conflicting results on the value of *PIK3CA* as a predictive biomarker for response to EGFR inhibitors, with some studies concluding that *PIK3CA* is an independent predictor of lack of response to anti-EGFR therapy, and others not reporting a correlation.^139–144^ This inconsistency may be due to differences in the frequency of *PIK3CA* mutations observed and their co-occurrence with *KRAS* mutations. A large retrospective analysis of 743 patients with mCRC revealed a negative correlation between *PIK3CA* mutation in exon 20 and response and survival following cetuximab treatment, which was not observed in patients with *PIK3CA* exon 9 mutations.^139^ However, since exon 20 mutations were only present in 3% of patients, further validation is needed to recommend routine use of *PIK3CA* testing in clinical decision-making.

Targeting *PIK3CA*-mutated tumors with agents inhibiting the PI3K/AKT/mTOR pathway is also being explored in mCRC. Therapeutic response to PI3K inhibitors in *PIK3CA*-mutated mCRC has been variable thus far, which may be partly explained by the intricacy of the PI3K signaling network, which intertwines with several other compensatory pathways, leaving opportunities for resistance.^145–148^ Thus, combination regimens including PI3K pathway inhibitors are underway (NCT04753203, NCT04495621, NCT02861300, and NCT03711058). In addition, absence or presence of co-occurring genetic alterations may impact the efficacy of PI3K inhibitors in *PIK3CA*-mutated mCRC. For example, several reports of patients with *PIK3CA* mutated solid tumors who achieved a partial response or prolonged stable disease following PI3K inhibitor therapy have reported co-occurring mutations in *ARID1*.^148,149^

Dysfunction in DNA damage response by mutations in the exonuclease domains of polymerase epsilon (*POLE*) and polymerase delta 1 (*POLD1*) leads to a hypermutated molecular phenotype and is thus also being explored as an independent marker for response to immune checkpoint inhibitors.^150^ A large study analyzing the mutation profile of 47,721 solid tumors found that mutations in *POLE* and *POLD1* were found in 7% of CRCs.^151^ In the overall population, 26% of patients with *POLE* and *POLD1* mutations were also MSI-H and mutated cases had a significantly higher TMB compared to wild-type cases. This study also reported an independent association between *POLE/POLD1* mutations and benefit from immune checkpoint inhibitors. Several clinical trials are underway, which plan to investigate the role of *POLE/POLD1* mutations on response to immune checkpoint inhibitors in mCRC (NCT031507061, NCT03435107, NCT03461952, and NCT03767075).

## Biomarker testing methodologies and reporting

### Testing methods and specimens

Many DNA-, RNA-, and protein-based assays are appropriate methods for evaluating the recommended mCRC biomarkers, if they are validated and performed by an accredited laboratory that follows quality guidelines, such as those set by the College of American Pathologists.^152^ Biomarker analysis in mCRC is increasingly being performed with multi-gene NGS panels across Canadian academic centers.^153^ This is likely due to the decreasing costs of NGS, and the many advantages to using multiplex testing in cancers with a rapidly evolving biomarker landscape, such as CRC.^154^ Using NGS, many genes and multiple classes of genomic alterations can be assessed simultaneously with greater sensitivity than other genomic testing approaches.^155^ In tumor sites where there are more than five actionable genomic biomarkers, NGS can be cost- and time-efficient, tissue-sparing, and can streamline the ordering and reporting of results for clinicians compared to sequential gene testing.^156,157^ Given the increasing number of relevant biomarkers for mCRC, transition to NGS panel testing should be considered.

Formalin-fixed, paraffin-embedded (FFPE) tissue is the preferred specimen for testing given that it is the most common tissue preservation method used in surgical pathology practice.^8^ Biomarker analysis using cytology specimens or different fixation protocols would require adequate validation. Either primary, metastatic, or recurrent tissue is an acceptable specimen for molecular biomarker evaluation, as several clinical studies have recorded concordance rates of over 90% for *RAS* and *BRAF* mutation status between primary and metastatic specimens.^158^

As the storage time for FFPE blocks increase, DNA/RNA quality and antigenicity can decrease, impacting the success of downstream molecular analyses. DNA fragmentation and cytosine to uracil deamination commonly occur after formalin-fixation and have been shown to increase with longer storage times, leading to a decrease in amplifiable DNA templates and G > A and C > T transitions, respectively.^159–162^ One study reported significant degradation of DNA extracted from the same FFPE blocks of surgically resected carcinomas of the lung, colon, and urothelial tract after 4–6 years of storage.^162^ This resulted in delayed target amplification of *KRAS* exon 2 with quantitative PCR, as well as a decrease in library yield and an increase in the number of single-nucleotide variants detected using NGS. The impact of increased FFPE tissue storage time on loss of antigenicity in the context of IHC assays is also well-documented, although the impact of storage time varies between antibodies used.^163,164^ Thus, the panel recommends that a new biopsy may be considered if an FFPE tissue block older than 5 years is the only available sample for testing. As biomarker analysis can still be successful using samples from older archival blocks, despite decreased DNA quality, it is also reasonable to attempt biomarker testing first and consider repeat biopsy if biomarker testing is unsuccessful or quality controls are suboptimal.

### Turnaround time and reporting

A rapid turnaround from sample acquisition to the reporting of biomarker results is necessary for preventing delayed treatment initiation. Meta-analyses covering studies across many tumor sites, including CRC, have reported an increased risk of death with every 4-week delay in initiation of curative treatment.^165,166^ Studies evaluating the impact of treatment delay in mCRC are less clear and may be confounded by the poorer prognostic profile of patients receiving accelerated treatment.^167^ A large retrospective study using data from the Taiwan Cancer Registry showed that an increase in the diagnosis to treatment interval for patients with mCRC, from less than 30 days to 31 to 150 days, resulted in a 37% increase in risk of death (HR 1.37, 95% CI: 1.28–1.47), when adjusted for other factors found to influence increase risk of death, including male gender, age >75 years, Charlson Comorbidity Index ⩾7, other catastrophic illnesses, lack of multidisciplinary team involvement, and treatment in a low volume center.^168^

There is also evidence to support the improved outcomes for patients with mCRC when biomarker-driven treatment is initiated in the first-line setting. In the KEYNOTE-177 trial evaluating mCRC patients with dMMR/MSI-H tumors, not only was the median PFS significantly longer for patients receiving pembrolizumab *versus* chemotherapy plus bevacizumab or cetuximab, but also PFS after next line of treatment (PFS2) was prolonged [median not reached *versus* 23.5 months (HR 0.63; 95% CI: 0.45–0.88)].^169^ Thus, testing workflow and procedures should be optimized to ensure that molecular biomarker testing results be reported to the oncologist by the time of the first consultation. Guidelines from international pathology associations and Canadian consensus publications recommend a maximum of 10 working days from sample receipt by the testing laboratory to generation of a summary report, with the report being sent to the referring oncologist within 24 h.^6,8,170–172^ For samples requiring send-out to a reference lab, the suggested turnaround time from specimen acquisition to arrival in the reference lab is three working days.^172^ Hospital systems should perform internal quality assurance assessments to evaluate whether turnaround time benchmarks are met. In cases where benchmarks are not met, strategies to improve turnaround time should be considered, which may include reflexive testing for all new CRC diagnoses, adjustments to workflow, and/or implementation of rapid biomarker testing methods.^8,173^

Reporting of biomarker testing results should conform to existing guidelines (American College of Medical Genetics, College of American Pathologists).^174,175^ Stating the testing method used, including details of which genomic alterations can be detected and the limitations of the test, is important as biomarker standards evolve over time. For example, the current recommendations for extended *RAS* mutation testing only include the analysis of missense mutations within exons 2, 3, and 4; however, emerging evidence on the utility of testing for RAS gene amplifications may result in its widespread adoption, and it would therefore be important to report. In addition, with the increased use of comprehensive genomic profiling by NGS, several genomic alterations with varying clinical significance may be detected. Thus, it will be important to report the likely pathogenicity of the identified variant as well as an interpretation section describing the therapeutic or prognostic implications of the results. The panel also recommends that in cases where the minimum required biomarkers for CRC are tested within a larger multi-gene panel, that genomic alterations identified outside the required genes be reported to the oncologist. This practice may be beneficial for diagnosis, staging, clinical research purposes, determining patient eligibility for clinical trials, and allowing patients compassionate access to therapies.

## Summary and future directions

Targeted therapies have increased the actionability of tumor molecular biomarkers in mCRC, particularly in earlier lines of treatment, and have brought the importance of timely molecular testing to the forefront. At minimum, the current biomarkers that must be evaluated to meet standard of care include mutational analysis of *NRAS*, *KRAS*, and *BRAF* genes, as well as determination of MMR/MSI status. In addition, *NTRK* fusions and *HER2* amplifications are actionable in mCRC and testing for these alterations should be considered as part of a multi-gene panel in all patients, or as a single-gene test in appropriately selected patients.

Ongoing clinical trials continue to push a biomarker-driven approach to the selection of therapy for CRC, with new biomarkers expected to be actionable in the coming years. Of particular interest are biomarkers of disease persistence and recurrence. Assays quantifying gene expression are being evaluated as prognostic classifiers for risk of disease recurrence in early-stage CRC. Thus far, assays including Oncotype Dx, ColoPrint, and ColDx have demonstrated some success in independently predicting risk of disease recurrence for patients with stage II/III CRC through gene expression profiling, whereas the ability to predict benefit of adjuvant chemotherapy has been less clear and requires further validation.^176–182^ Immunoscore, a unique scoring system evaluating the proportion of CD3+ and CD8+ immune cells within tumor samples, is also under investigation as a predictor for risk of recurrence in CRC.^183^

Liquid biopsies measuring circulating tumor DNA (ctDNA) are of great interest and show promising utility in the metastatic setting as a non-invasive alternative to biopsy-driven biomarker analysis, and they may provide insight on mechanisms of resistance to therapy, response to therapy, and early disease progression.^184–190^ Identification of ctDNA in the plasma of patients with localized CRC is being investigated, with great anticipation, as a surrogate marker of minimal residual disease to predict benefit from adjuvant chemotherapy in stage II CRC in clinical trials including COBRA (NCT0406810) and DYNAMIC-III (ACTRN12617001566325).^191^ Together, this highlights the growing importance of molecular testing in CRC and the need for centers to assess current testing workflow, equipment, and personnel, to ensure they are able to keep pace with the quickly evolving technologies necessary for practicing precision medicine in CRC.

## Declarations

**Ethics approval and consent to participate:** Not applicable.

**Consent for publication:** Not applicable.

**Availability of data and materials:** Not applicable.
